# Spreading Telehealth for Older Adults in Rural Areas Through Network of Geriatric Interprofessional Teams

**DOI:** 10.1093/geroni/igaa057.2885

**Published:** 2020-12-16

**Authors:** Steven Barczi, Megan Gately, Lauren Welch, Kathryn Nearing, Stephen Thielke, Camilla Pimentel, Laura Previll, Eileen Dryden

**Affiliations:** 1 University of Wisconsin, Madison; William S. Middleton Memorial Veterans Hospital, Madison, Wisconsin, United States; 2 Bedford VA Medical Center, Bedford, Massachusetts, United States; 3 William S Middleton VAMC GRECC, Madison, Wisconsin, United States; 4 University of Colorado Anschutz Medical Campus, Aurora, Colorado, United States; 5 VA Puget Sound Health Care System, Seattle, Washington, United States; 6 Edith Nourse Rogers Memorial Veterans Hospital, Center for Heallthcare Organization and Implementation research, Bedford, Massachusetts, United States; 7 Duke University School of Medicine, Durham, North Carolina, United States; 8 Center for Health Care Implementation Research (CHOIR), Bedford, Maryland, United States

## Abstract

Older adults living in rural areas have limited access to geriatrics interprofessional team care. In the Veteran healthcare system, geriatric teams such as geriatricians, nursing professionals, social workers, pharmacists and psychologists, located in urban areas link up with rural clinics to provide geriatric consultation remotely through clinical video telehealth and other means in the project GRECC Connect. Since its inception in 2014, the service has now grown to 16 geriatric teams offering consultation to over 100 clinic sites serving older rural Veterans. GRECC Connect delivered over 2,000 consultations in 2019, meeting complex care needs by identifying and linking geriatric services and management to patients with geriatric syndromes. The network of established geriatric teams, local champions and a shared Electronic Health Record facilitated the spread, while ongoing effort to build and maintain relationships between consultants and local rural provider teams and other community based services are important for ongoing success.

